# Cyclophosphamide-Induced Tolerance in Allogeneic Transplantation: From Basic Studies to Clinical Application

**DOI:** 10.3389/fimmu.2019.03138

**Published:** 2020-01-31

**Authors:** Koji Kato, Ario Takeuchi, Koichi Akashi, Masatoshi Eto

**Affiliations:** ^1^Department of Medicine and Biosystemic Science, Kyushu University Graduate School of Medical Sciences, Fukuoka, Japan; ^2^Department of Urology, Kyushu University Graduate School of Medical Sciences, Fukuoka, Japan

**Keywords:** tolerance, cyclophosphamide, graft-vs.-host disease, allogeneic hematopoietic stem cell transplantation, kidney transplantation

## Abstract

Immune tolerance against alloantigens plays an important role in the success of clinical organ and allogeneic hematopoietic stem cell transplantation. The mechanisms of immune tolerance to alloantigens have gradually been elucidated over time. Although there have been numerous reports to date on the induction of tolerance to alloantigens, the establishment of mixed chimerism is well-known to be crucial in the induction and maintenance of immune tolerance for either of the methods. Since the early 1980s, the murine system of cyclophosphamide (Cy)-induced tolerance has also been examined extensively. The present review focuses on studies conducted on Cy-induced immune tolerance. Clinical data of patients with allogeneic transplantation suggest that the posttransplant Cy method to induce immune tolerance has been successfully translated from basic studies into clinical practice.

## Introduction

Donor availability remains a limiting factor for success of allogeneic hematopoietic stem cell transplantation (allo-HSCT). A suitable human leukocyte antigen (HLA)-matched sibling or unrelated donor cannot be identified in time for about half of transplant recipients. On the other hand, an HLA-haploidentical donor can be identified rapidly in most cases. However, although rapid donor availability is the major advantage of HLA-haploidentical allo-HSCT, the obstacles of HLA-haploidentical allo-HSCT with a T-cell-replete graft include a high incidence of severe graft-vs.-host disease (GVHD), resulting in an increased incidence of non-relapse mortality at ~50% in early trials (1, 2). Certainly, *ex vivo* depletion of graft T cells reduces the risk of severe GVHD after HLA-haploidentical allo-HSCT; however, it is associated with an increased risk of engraftment failure and severe infections. Similarly, opportunistic infections which are associated with the suppression of cell-mediated immunity have now become pressing issues related to kidney transplantation, although the advances in immunosuppressants such as cyclosporine and tacrolimus have resulted in decreasing incidence of graft rejection in organ transplantation (3). These are as a result of non-specific immunosuppression, which suppresses the function of T cells in general.

To reduce the incidence of GVHD in HLA-haploidentical allo-HSCT through the donor-specific induction of immune tolerance in host or to avoid graft rejection in organ transplantation through the recipient-specific induction of immune tolerance against donor graft is the eventual goal for success of these allogeneic transplantation. This can be approached by selectively depleting alloreactive T cells; however, there is still no established method to achieve this goal. Recently, several research groups developed a method with high doses of cyclophosphamide (Cy) administered just after allogeneic transplantation (4, 5). In this article, we will provide a general outline of this topic, including a history of the basic research conducted to date.

## What Is Immune Tolerance?

Herein, we mainly describe immune tolerance to alloantigens (donor antigens) within the overall context of immune tolerance. First, however, we must discuss the induction and maintenance of tolerance to self-antigens. Tolerance against self-antigens is crucial in preventing the development of autoimmune diseases. Clonal deletion by eliminating autoreactive T cells has been proposed as the mechanism for the induction of tolerance. Tolerance has been clarified through the specific relationship between superantigens and certain Vβ segments of the T-cell receptor. In the late 1980s, clonal deletion in the thymus was shown in a mouse model with superantigens (e.g., Mls^a^ antigens), which can combine with major histocompatibility complex (MHC) antigen class II molecules and can respond strongly to T cells via the certain Vβ segments (e.g., Vβ6). In this mouse model with self-Mls^a^ antigens, specific Vβ6-positive T cells are eliminated in the periphery (6, 7). Indeed, these Vβ6-positive T cells were shown to be depleted during their differentiation in the thymus (central tolerance) (8). This was the first report of a method to explain the induction of self-tolerance through clonal deletion. Although central tolerance via clonal deletion is considered to be sufficient, they cannot control self-reactivity completely. Peripheral deletion mediated predominately via a Fas/FasL mechanism is one mechanism by which the immune system eliminates self-reactive T cells that escaped from central tolerance. Other mechanisms have been proposed for the induction and maintenance of self-tolerance. These include paralyzing autoreactive T cells (clonal anergy) and continuously suppressing autoreactive T cells by way of suppressor T cells. By these peripheral tolerances via regulatory T cells (Tregs) and cytokines, self-reactive T cells are rendered anergic even after encountering self-antigens outside of the thymus.

Immune tolerance against alloantigens plays an important role in the success of clinical organ and hematopoietic stem cell transplantation. There have been many reports of methods to date for the induction of tolerance to alloantigens (e.g., induction of immune tolerance in neonates, induction of tolerance using irradiation, induction of tolerance using monoclonal antibodies, and drug-induced immune tolerance). Although the establishment of mixed chimerism, in which donor cells are found at a certain rate in the recipient's body, is widely known to be crucial in the induction and maintenance of immune tolerance for either of the methods (9), MacDonald et al. demonstrated that the induction of immune tolerance in neonates was due to the intrathymic clonal deletion of alloantigen-reactive T cells (10). Especially after Starzl et al. reported that a microchimerism was established in some patients after liver transplantation in whom immunosuppressive treatment could be discontinued without the occurrence of graft rejection (11, 12), much work has been focused on how to induce immune tolerance by establishing chimerism in the field of clinical organ transplantation (13, 14). In addition, drug-induced immune tolerance with Cy was effective in xenotransplantation against B cells that produce xenoreactive antibodies (15). Thus, the mechanisms of immune tolerance to alloantigens have gradually been elucidated over time. Since the early 1980s, Professor Kikuo Nomoto's laboratory in the Department of Immunology, Medical Institute of Bioregulation, Kyushu University has extensively reexamined and developed a murine system of Cy-induced tolerance to show central and peripheral clonal deletion (4).

## Cyclophosphamide-Induced Immune Tolerance

Cy is a chemotherapeutic agent. Since Cy has been in clinical rotation for about 50 years, there is much experience to draw on for using this agent in the treatment of cancer and autoimmune diseases. Besides chemotherapeutic effects, Cy has immunosuppressive as well as immunomodulatory abilities. In 1963, Berenbaum and Brown first demonstrated the effects of Cy on the allogeneic response (16). Cy (200 mg/kg) was intraperitoneally administered to mice before or after an MHC-mismatched allogeneic skin graft (17). While control mice lost the allogeneic skin graft after ~14 days, mice treated with Cy revealed delayed skin graft rejection. When a single dose of Cy just after allogeneic skin grafting (day 0) was administered between days 0 and 4, it was shown to be more effective in prolonging graft survival compared to Cy use on day 6 or between days −4 and 0. Santos and Owens reported that Cy reduced the incidence and severity of GVHD when Cy was given on days 2, 3, and 5 after the infusion of allogeneic spleen cells in rats (18). From the extensive studies on the cells-followed-by-chemotherapeutic drugs system by many investigators, the optimal timing of chemotherapeutic drug use for the induction of tolerance is 1–4 days after antigen exposure; however, the different dose, timing, and cumulative exposure of Cy are critical for its efficacy in preventing GVHD (19). In addition, chemotherapeutic drugs such as 6-mercaptopurine, methotrexate, and 5-fluorouracil may be useful for promoting the success of the cells-followed-by-chemotherapeutic drug system, Cy is known to have the greatest tolerance induction potential among these chemotherapeutic drugs (20, 21). Treatment of Cy with other immunosuppressive drugs such as steroids, cyclosporine, and tacrolimus before or together with the allogeneic cell infusion impaired the development of the tolerance induction because the cell proliferation was inhibited as a result of these drugs being used for pretreatment (22).

**Figure 1:**
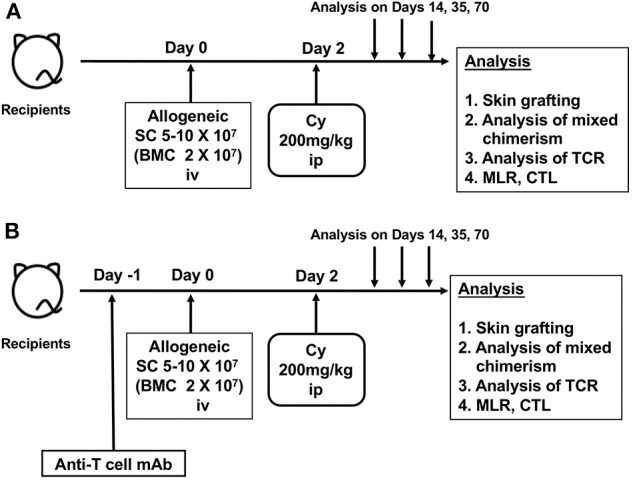
The schema of cyclophosphamide-induced tolerance model. **(A)** Original protocol of cyclophosphamide-induced tolerance model. **(B)** Modified protocol using anti-T-cell monoclonal antibody. SC, spleen cell; BMC, bone marrow cell; Cy, cyclophosphamide; mAb, monoclonal antibody; ip, intraperitoneal injection; iv, intravenous injection; MLR, mixed lymphocyte reaction analysis; CTL, cytotoxic T-lymphocyte analysis; TCR, T-cell-receptor analysis.

Based on these results, the Nomoto's laboratory developed a method, the so-called “cells-followed-by-Cy system” for inducing tolerance to allogeneic grafts (4, 23). Here, the method of the Cy-induced immune tolerance that we have reported on to date consists of a very simple process of administering 5–10 × 10^7^ allogeneic spleen cells (bone marrow cells were later added) intravenously 2 days before intraperitoneally administering Cy at a dose of 150–200 mg/kg (Figure 1A). The most distinctive feature is the administration of Cy after rather than before the administration of alloantigens (24–26). Furthermore, the “cells-followed-by-Cy system” in combination with using anti-T-cell monoclonal antibody and low-dose total body irradiation (TBI) on day −1 could induce a profound tolerance with sustained mixed chimerism to skin or other solid organs in various mouse combinations with differing MHCs and minor antigens (5, 27–29) (Figure 1B). Thus, through the extensive efforts of basic researches, this “cells-followed-by-Cy system” has been also referred to as “posttransplant Cy (PTCy)” in the clinical field of allo-HSCT and solid organ transplantation.

## Mechanisms of Cyclophosphamide-Induced Immune Tolerance

**Figure 2:**
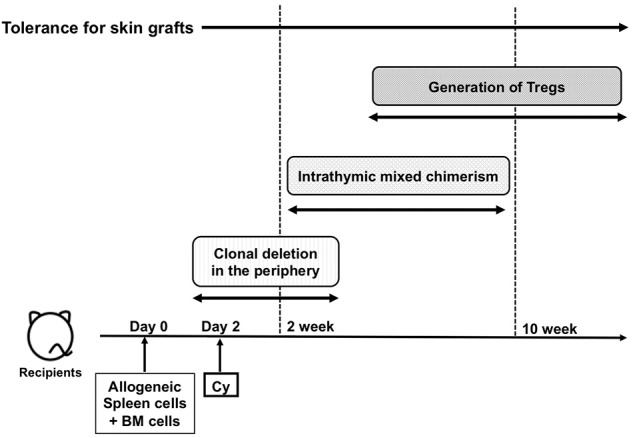
The flow of the three major mechanisms of cyclophosphamide-induced tolerance. Cy, cyclophosphamide; BM, bone marrow; PBSCT; Treg, regulatory T cells.

Using the aforementioned correlations between Mls^a^ antigens and Vβ6-positive T cells, the Nomoto's laboratory investigated the mechanism of immune induction and maintenance of tolerance to alloantigens through Cy-induced immune tolerance and identified the following three distinct and sequential mechanisms in MHC-matched setting. The first mechanism was the deletion of alloantigen-stimulated T cells after Cy treatment in the periphery (30, 31). The second mechanism was intrathymic clonal deletion of donor-reactive T cells, which was strongly associated with mixed chimerism in the thymus during the maintenance phase. The third mechanism was the generation of suppressor T cells in the late stage of the tolerance. The flows of the three mechanisms over time are summarized in Figure 2.

### Clonal Deletion of Donor-Reactive T Cells During the Induction of Tolerance

When AKR/J (H-2k, Mls^a^, Thy-1.1) is used as the donor and C3H/He (H-2k, Mls^b^, Thy-1.2) is used as the recipient, long-term engraftment of AKR/J skin allografts can be induced through Cy-induced immune tolerance (32). When AKR/J splenocytes were administered to recipient C3H/He and Cy was administered 2 days later, the number of CD4-positive Vβ6-positive T cells with strong reactivity to donor Mls^a^ antigens selectively decreased from an early stage and were remarkably decreased at 5 weeks after Cy administration. No such changes were observed in control Vβ8-positive T cells, indicating that the findings were specific to Vβ6-positive T cells. In addition, since CD8-positive T cells were less reactive to Mls^a^ antigens in Vβ6-positive T cells, a response similar to the one observed in CD4-positive T cells was not seen. These results indicate that alloreactive T cells are selectively eliminated after Cy in the periphery. The clonal deletion mechanism acts on mature T cells, which are outside of the bone marrow and thymus. In addition, clonal deletion at the time of induction of tolerance was also observed in recipient-reactive T cells from treated donors, and an involvement in the prevention of GVHD was also revealed through an evaluation of the transition of Vβ3-positive T cells from donor AKR/J reactive to Mls-2^a^ antigens of recipient C3H/He.

### Intrathymic Mixed Chimerism in the Maintenance Phase

In AKR/J → C3H/He combinations, while Thy-1.1-positive T cells from donor AKR/J were observed in peripheral lymph nodes beginning immediately after the induction of tolerance, Thy-1.1-positive T cells from the donor AKR/J were not observed in the thymus of recipient C3H/He on day 14 after Cy administration, and Vβ6-positive T cells reactive to donor Mls^a^ antigens were found at normal levels (32). However, by day 35 after the administration of Cy, Thy-1.1-positive T cells from donor AKR/J were found in the thymus of recipient C3H/He, suggesting that the hematopoietic cells in donor AKR/J splenocytes had differentiated and matured in the thymus of recipient C3H/He. In the thymus that was in such a chimeric state, there was clonal deletion of Vβ6-positive T cells reactive to donor Mls^a^ antigens. This indicates that chimerism is also established at the antigen-presenting cell level in recipient thymus. Clonal deletion of Vβ6-positive T cells was observed in either CD4- or CD8-positive mature thymic T cells, unlike the aforementioned peripheral clonal deletion, to allow for the negative selection of CD4- and CD8-positive immature thymocytes in the thymus (10, 33).

### Generation of Tolerogen-Specific Regulatory T Cells in the Late Maintenance Phase

Although the clonal destruction of alloreactive T cells is thought to be the dominant mechanism of Cy-induced tolerance, it is insufficient to explain peripheral tolerance in the Cy-induced tolerance system. Tregs also have an important role in Cy-induced tolerance system (34–37). In DBA/2 (H-2^d^, Mls^a^) → BALB/c (H-2^d^, Mls^b^) combinations, intrathymic T-cell chimerism disappeared by day 100 following the induction of tolerance in some recipient BALB/c mice and, as a consequence, the intrathymic clonal deletion of Vβ6-positive T cells reactive to donor Mls^a^ antigens was also disrupted, with the regeneration of Vβ6-positive T cells observed in peripheral lymph nodes (38). This suggests that chimerism was also lost at the level of antigen-presenting cells in the thymus. Nevertheless, the skin allografts remained engrafted. Therefore, the presence or absence of the involvement of Tregs was evaluated as a mechanism of maintaining tolerance at this stage. The adoptive transfer of splenocytes from mice engrafted with donor skin allografts into syngeneic mice irradiated with low doses of radiation followed by the grafting of donor skin allografts on the following day revealed the presence of donor antigen-specific Tregs (39), with CD8-positive T cells being predominantly found in this combination. Tregs were insufficient at day 14 to prevent skin allograft rejection upon transfer. In addition, the presence of CD4-positive Tregs has also been observed among mice differing only in terms of class II antigens (40).

Because MHC-matched murine skin-allografting models with Mls antigens were highly contextual, the exact association of these three mechanisms to how PTCy prevents GVHD in allo-HSCT still remains unclear. Donor Tregs, which are resistant to PTCy via aldehyde dehydrogenase expression, are necessary for protection against GVHD (35, 37). A recent report showed that PTCy did not eliminate alloreactive T cells and the thymus was not necessary for efficacy of PTCy in T-cell replete, MHC-haploidentical, murine allo-HSCT model (B6C3F1**→**B6D2F1), whereas PTCy impaired the function of alloreactive T cells and the rapid recovery of Tregs played an important role in suppressive mechanisms of GVHD (36). In four other models including one of the same MHC-matched strain combinations as used in the skin allografting models, PTCy also did not eliminate alloreactive T cells. Rather, PTCy induced alloreactive T-cell functional impairment over time through the suppressive mechanism with rapid recovery of alloantigen-specific Tregs (36). In addition to the role of Tregs, clonal anergy was suggested to be involved in the maintenance of tolerance in the late phase of maintenance (41, 42), although this did not appear in allo-HSCT (36). In the early stage of induction of Cy-induced tolerance, observing higher chimerism of donor-derived cells in the recipient periphery was also found to be important in inducing a higher level of tolerance (43). The differential influence of Cy on each subset of T cells has been also reported. Among T cells spared by Cy treatment, naive-derived memory stem T cells (44–47) that can differentiate into various memory T cells are the most abundant T-cell population in the early period following PTCy haploidentical allo-HSCT and play an important role in immune reconstitution in the long term after transplantation (48, 49). Therefore, further understanding of tolerance induction by PTCy can be put in perspective in future studies.

## Clinical Application of Cyclophosphamide-Induced Immune Tolerance

The outline of the historical background on underlying mechanisms in Cy-induced immune tolerance has hereby been provided. Of note, however, Cy-induced tolerance is now also receiving great attention in two clinical fields: allo-HSCT and kidney transplantation.

### Clinical Application in Allo-HSCT

As previously described, a characteristic feature of the PTCy method is the fact that Cy is given after rather than before the administration of alloantigens. Especially in the field of HLA-haploidentical allo-HSCT, this method has garnered significant attention with respect to good engraftment and low incidence of GVHD. The Johns Hopkins University group has previously successfully achieved tolerance and mixed hematopoietic chimerism in mice treated with fludarabine (Flu) and 200 cGy TBI, transplanted with 10 million marrow cells, and given Cy 200 mg/kg intraperitoneally on day 3 (5). This method reduced the incidence and severity of GVHD in an MHC-mismatched combination. These promising results provided the rationale to conduct a clinical trial of PTCy HLA-haploidentical allo-HSCT for patients with poor prognoses for hematological malignancies (50–52) and non-neoplastic hematological diseases (53).

Based on these results, a phase I/II clinical trial of haploidentical bone marrow transplantation for hematological malignancies was initiated in 1999. Thirteen patients with hematological malignancies received conditioning with Flu (30 mg/sqm/day from days −6 to −2) and TBI (200 cGy on day −1). All patients received Cy at a single dose of 50 mg/kg on day 3 with tacrolimus and mycophenolate mofetil from day 4 as a GVHD prophylaxis (51). Of the first three patients, two conditioned without Cy developed engraftment failure in the phase I portion. Therefore, Cy was added to the conditioning regimen at a total dose of 29 mg/kg given on days −5 and −6 in the next 10 patients, and 8 could achieve engraftment. Acute GVHD developed in six of these eight engrafted patients during the phase II portion and responded well to treatment. Results of the first 13 patients were reported as a proof-of-principle report in 2002 (51). Clinical outcomes of 68 patients in phase II trials were also reported in 2008 (52). In this trial, the dose of administered Cy was modified by increasing the total dose of PTCy to 100 mg/kg given over days 3 and 4 to decrease in the incidence of GVHD. Among 68 patients, 40 patients received the total dose of PTCy with 50 mg/kg on days +3 and +4, and 28 patients received 50 mg/kg on day +3. Primary engraft failure occurred in 13% of patients. The median times for neutrophil and platelet recovery were 15 and 24 days, respectively. There was no difference in the incidence of severe acute GVHD between one or two doses of PTCy with 34% of cumulative incidences being of grades II–IV and 6% being of grades III–IV acute GVHD. The cumulative incidences of non-relapse mortality and relapse at 1 year were 15 and 51%, respectively.

**Figure 3:**
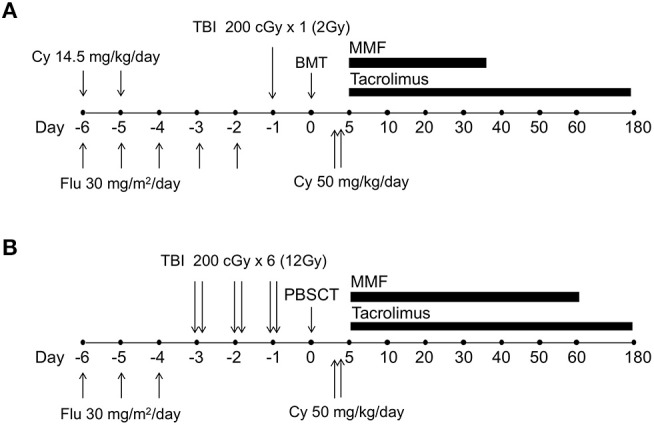
The schema of human leukocyte antigen (HLA)-haploidentical transplantation with posttransplantation cyclophosphamide. **(A)** The schema of non-myeloablative, HLA-haploidentical bone marrow transplantation with posttransplantation cyclophosphamide, which was originally developed by the Johns Hopkins University group. **(B)** One example schema for myeloablative, HLA-haploidentical peripheral blood stem cell transplantation with posttransplantation cyclophosphamide. Cy, cyclophosphamide; Flu, fludarabine; TBI, total body irradiation; BMT, bone marrow transplantation; PBSCT, peripheral blood stem cell transplantation; MMF, mycophenolate mofetil.

Thus, PTCy as GVHD prophylaxis has been developed initially for haploidentical bone marrow transplantation after non-myeloablative conditioning (Figure 3A); however, myeloablative conditioning or peripheral blood stem cells as the graft source have been successfully used in several studies (54–56). Among the many platforms used with PTCy, an example of a myeloablative approach with peripheral blood stem cells is shown in Figure 3B. In addition, recent studies demonstrated that PTCy could be applied even in HLA-matched allo-HSCT, and donor type might no longer be a significant predictor in the era of PTCy (57–59). Consequentially, allo-HSCT with PTCy has spread rapidly worldwide.

### Clinical Application to Kidney Transplantation

Clinical trials to induce renal allograft tolerance with allogeneic stem cell have been reported from three centers: Northwestern University, Stanford University, and Massachusetts General Hospital in the United States (60). Aside from the methods inducing tolerance by PTCy, Stanford University and Massachusetts General Hospital groups have been using their specific approaches to induce renal allograft tolerance (61). Researchers at Stanford University group used allo-HSCT with total lymphoid irradiation and rabbit antithymocyte globulin to induce mixed chimerism. In their experience, durable or transient chimerism was induced in HLA-matched transplant recipients, and immunosuppressive agents were withdrawn in ~70% of the patients; however, induction of chimerism has been difficult in HLA-mismatched transplant recipients, and no recipients has achieved complete discontinuation of immunosuppression. The Massachusetts General Hospital group developed the conditioning regimen for HLA-mismatched kidney transplantation, which included Cy (60 mg/kg on days −5 and −4), thymic irradiation, anti-CD2 monoclonal antibody, and posttransplant calcineurin inhibitors administration (13, 14). In a revised regimen, low-dose TBI replaced Cy, and rituximab was added. Of the 10 recipients enrolled in the studies, all developed transient mixed chimerism and immunosuppression was discontinued in eight patients. After a follow-up period of 7–14 years, four patients still could discontinue immunosuppression completely (62).

**Figure 4:**
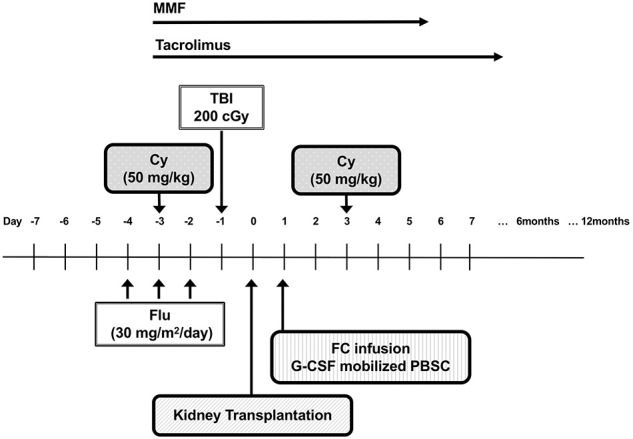
The schema of living-donor kidney transplantation with posttransplantation cyclophosphamide. Cy, cyclophosphamide; Flu, fludarabine; TBI, total body irradiation; FCs, facilitating cells; MMF, mycophenolate mofetil002E.

The Northwestern University group reported results from a recent clinical trial involving patients undergoing living-donor kidney transplantation using the regimen shown in Figure 4, where stable donor-derived chimerism was able to be induced in 19 out of 31 recipients and immunosuppressive agents could also be discontinued (60, 63). In this report, patients were transplanted from six of six HLA-matched related to zero of six HLA-matched unrelated donor. Twelve subjects had unrelated and 19 had related donors. The patients are conditioned with Flu (30 mg/sqm/day on days −5, −4, −3), Cy (50 mg/kg on day −3), low-dose TBI (200 cGy on day −1) followed by living-donor kidneys transplant (day 0). The patients received donor bone marrow after living-donor kidneys were transplanted. Regarding stem cell transplantation, a population of so-called facilitating cells from donor bone marrow cells was enriched and used, after which point, Cy was administered at a dose of 50 mg/kg, 2 days later. Facilitating cells are identified as CD8-positive and α*βγδ* T-cell receptor-negative donor bone-marrow-derived cells that promote allogeneic stem cell reconstitution. In the recent report, two patients developed GVHD, and two patients experienced renal allograft losses. Although not yet a perfect induction of tolerance, the ability to discontinue immunosuppressive drugs in two-thirds of patients can be considered a great achievement. Combined hematopoietic stem cell and kidney transplantations have shown efficacy and safety, as well as validated the proof of principle of inducing tolerance by PTCy (64–67).

## Conclusion

In this report, we outlined the history of Cy-induced immune tolerance. Collectively, clinical data suggest that the PTCy method for the induction of immune tolerance has been successfully translated from basic studies to clinical application. So far, the data have been adequately encouraging us to further develop PTCy approach in future studies.

## Author Contributions

KK, AT, KA, and ME wrote the manuscript and created the figures. All authors critically reviewed the manuscript and read and approved the final version of the manuscript.

### Conflict of Interest

The authors declare that the research was conducted in the absence of any commercial or financial relationships that could be construed as a potential conflict of interest.
